# Information Transfer and Multifractal Analysis of EEG in Mild Blast-Induced TBI

**DOI:** 10.1155/2021/6638724

**Published:** 2021-04-06

**Authors:** Todd Zorick, Katy D. Gaines, Gholam R. Berenji, Mark A. Mandelkern, Jason Smith

**Affiliations:** ^1^Department of Psychiatry, Harbor-UCLA Medical Center and UCLA Geffen School of Medicine, USA; ^2^Neuro Health Inc, University of California, Irvine, USA; ^3^Greater Los Angeles VA Department of Nuclear Imaging, University of California, Irvine, USA; ^4^Department of Physics, University of California, Irvine, USA; ^5^The Boeing Company, USA

## Abstract

Mild, blast-induced traumatic brain injury (mbTBI) is a common combat brain injury characterized by typically normal neuroimaging findings, with unpredictable future cognitive recovery. Traditional methods of electroencephalography (EEG) analysis (e.g., spectral analysis) have not been successful in detecting the degree of cognitive and functional impairment in mbTBI. We therefore collected resting state EEG (5 minutes, 64 leads) from twelve patients with a history of mbTBI, along with repeat neuropsychological testing (D-KEFS Tower test) to compare two new methods for analyzing EEG (multifractal detrended fluctuation analysis (MF-DFA) and information transfer modeling (ITM)) with spectral analysis. For MF-DFA, we extracted relevant parameters from the resultant multifractal spectrum from all leads and compared with traditional power by frequency band for spectral analysis. For ITM, because the number of parameters from each lead far exceeded the number of subjects, we utilized a reduced set of 10 leads which were compared with spectral analysis. We utilized separate 30 second EEG segments for training and testing statistical models based upon regression tree analysis. ITM and MF-DFA models both generally had improved accuracy at correlating with relevant measures of cognitive performance as compared to spectral analytic models ITM and MF-DFA both merit additional research as analytic tools for EEG and cognition in TBI.

## 1. Introduction

Human EEG recordings have been utilized for clinical and research purposes since the 1930s, but much is still unknown about the underlying neuronal dynamics responsible for scalp-recorded electric potential changes as a function of time. Recently, many lines of investigation in neuroscience and statistical physics have converged to raise the hypothesis that the underlying pattern of neuronal activation which results in EEG trace recordings is nonlinear, with scale-free dynamics [1, 2], while EEG signals themselves are nonstationary and also show scale-free dynamics [3]. Therefore, traditional statistical methods of EEG analysis (e.g., Fourier Transform and frequency-averaged spectral analysis) may not be the most appropriate means to analyze EEG signals, since these techniques would miss many properties inherent in nonstationary signals with scale-free dynamics [2].

In an attempt to take these considerations seriously, we have demonstrated that two separate approaches derived from physics of time series analysis have the potential to be improvements over existing techniques (including spectral analysis), summarized below.

*Multifractal detrended fluctuation analysis* (*MF-DFA*): we have shown that human EEG signals are well-modeled as a multifractal time series [4]. We have also shown that within subjects, sleep stages are clearly distinguished from waking via multifractal detrended fluctuation analysis (MF-DFA; [4]). Additionally, in a study of patients with Alzheimer's disease, mild cognitive impaired, and age-matched controls, MF-DFA EEG analysis was shown to predict cognitive function scores in an out-of-sample EEG dataset [5]

*Information transfer modeling* (*ITM*): we have also demonstrated that human sleep EEG stages are readily differentiated from waking EEG within subjects by means of ITM analysis [6]. ITM specifically models EEG voltage fluctuations as an “information transfer device” [7, 8], as a way of characterizing a generalized nonequilibrium thermodynamic process, via estimation of the information transfer constant as a function of time. While our previous publication described the within-lead ITM procedure, in this manuscript, we provide an additional novel application to between-lead ITM analysis and take advantage of both spatial and temporal information transfer patterns to provide a more comprehensive understanding in multilead EEG (see methods for complete details)

To date, neither MF-DFA nor ITM has been directly compared to spectral analysis (via Fourier Transform) in their ability to generate EEG-derived parameters that correlate with clinically meaningful data. In this manuscript, we show data from a study performed on a group of individuals with a history of mild blast-induced traumatic brain injury (mbTBI), with neuropsychological testing for executive functioning (D-KEFS Tower test [9]), in conjunction with resting state EEG recordings.

TBI is a devastating consequence of multifactorial causes of brain damage, which results in considerable morbidity to affected individuals. In 2014, about 2.87 million TBI-related emergency department (ED) visits, hospitalizations, and deaths occurred in the United States [10]. Over the span of eight years (2006–2014), age-adjusted rates of TBI-related ED visits increased by 54% [10]. The peak of incidence of TBI in the general population is in males aged 15 to 24, and it has been estimated that several million people in the U.S. alone may suffer from resultant lifelong cognitive and physical impairment [11].

In U.S. veteran populations, TBI presents a considerable ongoing clinical challenge. Operation Enduring Freedom (Afghanistan)/Operation Iraqi Freedom-returning veterans suffered from high rates of TBI primarily due to concussive blast injuries [12]. Nearly 7% of veterans seeking U.S. Veterans Administration (VA) treatment in 2009 were diagnosed with TBI; of those, 89% had a cooccurring psychiatric disorder (73% PTSD), and 70% suffered from a chronic somatic pain disorder [12]. These proportions were far higher than those in veterans without TBI; all told, veterans with TBI incurred annual medical costs more than three times greater than those without ($5831/year vs. $1487/year in 2009). Therefore, TBI and the resultant increased psychiatric, and medical comorbidities represent a large ongoing challenge to military and veteran health care providers.

Many serious diagnostic and treatment-specific problems currently limit the ability of clinicians and health care organizations to treat TBI, as there are no single specific biomarkers for neurocognitive impairment or functional capacity limitations associated with TBI [12]. The gold standard for diagnosis and treatment of TBI is an expensive, intensive battery of neuroimaging, medical, and neuropsychiatric testing to arrive at a diagnosis and that utilizes a complex diagnostic process to determine impressions of the patient's level of functional capacity [12]. Additionally, existent screening tools designed to assess for TBI are very nonspecific and frequently provide positive screens for patients with no history of brain injury [13]. One of the primary areas of cognitive dysfunction that individuals with TBI exhibit post injury is the executive, which is responsible for important life functions, such as planning and organizing, following through with tasks, analyzing complex information, and modulating emotions. Therefore, there exists an urgent need to develop specific diagnostic tests for executive function deficits in TBI, both to aid in diagnosis and treatment.

The overall aims of this preliminary study were to compare ITM-, MF-DFA-, and FT-derived indices from resting EEG tracings in their ability to correlate with clinically meaningful measures of executive function in a group of twelve participants with mbTBI without neuroanatomical lesions (dataset originally described in [14]). We hypothesized based upon theoretical and practical considerations that both MF-DFA and ITM would prove superior to FT as an EEG-derived correlate for executive functioning.

## 2. Materials and Methods

### 2.1. Summary of Experimental Design

Participants (*n* = 12) who previously participated in a Greater Los Angeles VA Healthcare System (GLAVAHS) research study of PET scanning as a potential diagnostic tool for mbTBI (“Regional cerebral metabolism in blast-induced mild TBI”, Dr. Berenji, P.I. [14]) were invited back for collection of resting-state EEG (~5 minutes) and cognitive testing via a test not utilized in the original study. Participants eligible for the study had a history of mild blast-induced TBI incurred during their military service within five years of study entry, with no identifiable structural lesions. The inclusion criterion for the study was completion of the VA research study “Regional cerebral metabolism in blast-induced mild TBI.” The exclusion criteria were any indication of suicidal ideation (i.e., as assessed by question 9 on the Beck Depression Inventory, Revised (BDI-II)) or inability to participate in the EEG collection or cognitive testing. Per VA research guidelines, participants were compensated for their participation. All procedures described in this study were approved by the GLAVAHS IRB, and informed consent obtained from all subjects.

### 2.2. Behavioral and Cognitive Measures

*Beck Depression Inventory*, *Revised* (BDI-II [15]) is a 22-item measure of depressive symptomatology, widely used in psychological research and practice.

#### 2.2.1. Delis-Kaplan Executive Function System (D-KEFS): Subtest—Tower Test

The D-KEFS is designed to assess the key components of executive functions believed to be associated primarily with the frontal lobe. The Tower test measures learning of rules, visuo-spatial planning, inhibition of impulsive responses, perseveration, and the ability to maintain and establish an instruction-based set [9]. The time-per-move ratio score (TPM) was utilized as the primary outcome measure, as deficits in the cognitive ability to plan moves are largely independent of motor function [16] and are implicated as a sensitive indicator of frontal lobe dysfunction [17, 18]. Secondary outcome measures including total achievement score (ACH), mean first move time score (FMT), move accuracy ratio score (MAR), and rule violations per item ratio score (RVPI), and total rule violation (TRV) score was also assessed.

*Montreal Cognitive Assessment* (MOCA) is designed to provide an accurate assessment for mild to severe cognitive impairment associated with dementia such as Alzheimer's disease or moderate to severe brain injury [19].

### 2.3. EEG Methods

#### 2.3.1. EEG Acquisition

We collected five minutes of resting-state EEG on participants utilizing a 64-channel EEG cap via the NetStation EEG system (Electrical Geodesics, Eugene, OR). Participants' EEG activity was continuously recorded during 5 minutes while patients were instructed to sit quietly with their eyes closed. EEG data were sampled at 250 Hz with filter settings of 0 to 100 Hz in DC acquisition mode. 64 cap-mounted, equidistant sintered Ag-AgCl electrodes were positioned via manufacturers' instructions for use. EEG data were processed offline using NetStation EEG software, using a right mastoid reference electrode. However, given time constraints for participant contact and limited equipment availability, many of the recorded leads demonstrated elevated levels of impedance (~40% per participant on average), which likely impaired the overall quality of recorded EEGs. Nonetheless, all leads were utilized for further analysis, as a test of likely “real world” application of the EEG analytic techniques as described.

#### 2.3.2. EEG Analysis

We chose two separate 30 s epochs from each subject (one from 90 to 120 s of recording and one from 210 to 240 s of recording) for further analysis, using all 64 leads irrespective of the possibility of movement artifacts or poor impedance. These epochs were used separated for training and testing EEG data in order to, respectively, train and test our statistical models (see below).

#### 2.3.3. MF-DFA EEG Analysis

64 lead digital EEG tracings (30 s in each of two epochs as above) were analyzed using MF-DFA as described [4], using right mastoid as a secondary reference electrode. The following parameters were extracted from each lead: mean, minimum, maximum Hölder exponents, and width of Hölder exponent spectrum for further analysis.

#### 2.3.4. ITM EEG Analysis

We performed both within-lead ITE analysis as described [6] but further characterize a between-lead EEG ITM analysis herein. Briefly, given appropriate constraints, virtually any physical system can be considered from the point of view of information transfer, whereby information flows from a source (*q*) to a destination (*u*) [7, 8]. From information theory, therefore, the information flow (*I*) must obey the inequality:

(1) $$\begin{array}{c} I_{u}\leq I_{q}. \end{array}$$

This is because information received cannot exceed information transmitted. Following this, Fielitz and Borchardt derived equations describing information transfer relationships for several different physical systems [7, 8]. Relevant to EEG, ITM predicts that there will be an information transfer constant (*κ*) for each instant such that voltage changes (Δ*V*) are proportional to corresponding time intervals (Δ*t*):

(2) $$\begin{array}{c} \left|∆V\right|\sim \left|∆t\right|\kappa . \end{array}$$

EEG is a time series of voltage readings *V*(*t*), where *t* = 1, 2, ⋯*n*, (length of series) for each value of *t* up to *n* − Δ*t*, given a time interval Δ*t*. In this manner, the value of the information transfer constant (*κ*) for each instant can be calculated:

(3) $$\begin{array}{c} \kappa_{t}=\frac{\log\left(V\left(t+\Delta t\right)-V\left(t\right)\right)}{\log\left(\Delta t\right)}. \end{array}$$

Therefore, each segment of EEG would be characterized by a series of information transfer constant ratios, for different values of the time interval Δ*t* (i.e., 1, 2, 4, 8, and time steps):

(4) $$\begin{array}{c} \kappa_{\left(\text{segment},\Delta t\right)}=\overset{n-\Delta t}{\underset{t=1}{\sum }}\kappa_{t}. \end{array}$$

Mean information transfer constants as a function of time interval Δ*t* can provide an accurate description of sleep staging from a single lead [6].

Principles of ITM can also be extended to between-lead information flow as a function of time. For two EEG leads (e.g., l1 and l2) placed on different parts of the scalp, a measure of between-lead information flow can be defined by the information transfer constant ratio:

(5) $$\begin{array}{c} \kappa_{\text{itcr}}=\frac{\kappa_{l1}}{\kappa_{l2}}. \end{array}$$

This implies that for a given time interval Δ*t*, the between-lead information flow for two leads (l1 and l2) can be assessed by the following relation:

(6) $$\begin{array}{c} \kappa_{\text{itcr},\Delta t}=\frac{\log\left(\left|V_{l1}\left(t+\Delta t\right)-V_{l1}\left(t\right)\right|\right)}{\log\left(\left|V_{l2}\left(t+\Delta t\right)-V_{l2}\left(t\right)\right|\right)}. \end{array}$$

In a manner similar to the single-lead ITM above (see equations (2)–(4); [6]), the most meaningful way to utilize the information transfer constant ratio as a function of time interval would be as a mean value over a given length (*N*) of EEG (e.g., 30 s), with a series of different time intervals (e.g., ranging from 4 ms to 4 s in natural logarithmic steps).

(7) $$\begin{array}{c} \text{Segment Mean }\kappa_{\text{itcr},\Delta t}=\frac{\sum_{1}^{N-\Delta t}\kappa_{\text{itcr},\Delta t}}{N-\Delta t}. \end{array}$$

Given that the combination of within- and between-lead ITM analysis provides for an extremely large number of possible combinations (i.e., 5 time steps per lead, along with 5 time steps for each between-lead ITM combination, >20,000 possible data points for all combinations of 64 leads with only 12 subjects), this would prevent the practical use of data mining techniques. Therefore, we chose a subset of 10 electrodes covering the majority of the scalp to keep the number of data points down to a reasonable number amenable to subsequent analysis. The choice of 10 leads for ITM analysis was informed by balancing the desire to adequately sample the entire scalp with keeping the number of leads as few as possible to avoid underperformance of statistical modeling. The leads used were the following (see Electrical Geodesics manual for exact locations): leads 3 and 56 (right frontal area), leads 9 and 19 (left frontal area), 6 and 36 (anterior and posterior midline, respectively), 40 and 46 (right parietal), and 26 and 31 (left parietal).

#### 2.3.5. Spectral Analysis

We utilized the R program “spec.pgram” to perform a fast Fourier Transform on the same EEG utilized for MF-DFA and ITM, separately [20]. We extracted total spectral power for the following bandwidths: alpha (8-14 Hz), beta (16-31 Hz), delta (0.1-4 Hz), gamma (32-50 Hz), and theta (4-7 Hz) for each 30 s segment of each lead.

### 2.4. Statistical Methodology

As above, the two separate 30 s EEG segments (either all 64 leads for MF-DFA and matched FT or subset of 10 leads for ITM and matched FT analysis) were analyzed by each method listed above, respectively. The method of Classification and Regression Trees (CART) was used on the first (training) EEG parameter set, with a minimum split of 4, using the R program “rpart” [21], designed to follow CART [22] closely. The rpart model utilized output from the D-KEFS Tower test subscores to make a regression model with the EEG training data parameters (1st set of EEG-derived parameters). In order to minimize bias-variance and overfitting problems, we utilized 10-fold cross validation for choosing an optimal complexity parameter for each CART model on the training dataset. Following [23], we chose complexity parameters based upon the smallest CART model within 1 standard error of the model with the minimum cross-validation error, and used these tuned models for predicting cognitive function from the test datasets. The trained, pruned rpart model for each subscore and EEG training data parameters was then used to predict the subscore from the second EEG test data parameters for each analysis. For correlations between the predicted EEG test data parameters and the actual subscores, Pearson's moment correlation testing was performed using R. CART tree plots in Supplementary Figure 1 were drawn using the R “rpart.plot” package [24]. We choose CART for two main reasons, firstly, that CART models are straightforward in their physiological interpretation, such that precise EEG-based parameters that lead to successful models are readily identified. Secondly, traditional machine learning paradigms such as Support Vector Machines analysis produced such high correlations in similar training/testing paradigms that there were no observable differences between the different methods tested (data not shown; parameters tuned with 10-fold cross validation on training data).

## 3. Results

### 3.1. Demographic and Clinical Characteristics of the TBI Participants

The subjects had a mean age of 30.8 ± 2.7 years of age. The sample was mostly male (10 subjects), mostly racially white (9 subjects; 2 black subjects), with a substantial proportion of Hispanic ethnicity (5 subjects), largely representative of the veteran population in the greater Los Angeles metropolitan region. There was no association between age, gender, race/ethnicity, BDI score, or MOCA score and any Tower test subscores (Table 1). All participants had perfect 30/30 MOCA scores, whereas there was significantly more variability in BDI scores and Tower test subscores amongst participants on the procedure day (Table 1). In addition to a diagnosis of a mild blast-induced TBI, all participants were also independently diagnosed (using the Structured Clinical Interview for Dsm-5 Disorders (Scid-5-cv): Research Version) with posttraumatic stress disorder as a result of their military combat experience. Despite elevated levels of depressive symptoms seen across subjects (BDI score 21 ± 8.9, Table 1), only four subjects carried a previous diagnosis of major depression, and none were taking psychiatric medications at the time of the study.

### 3.2. MF-DFA Differs from FT on Ability to Predict Test EEG-Derived Tower Test Scores

**Figure 1:**
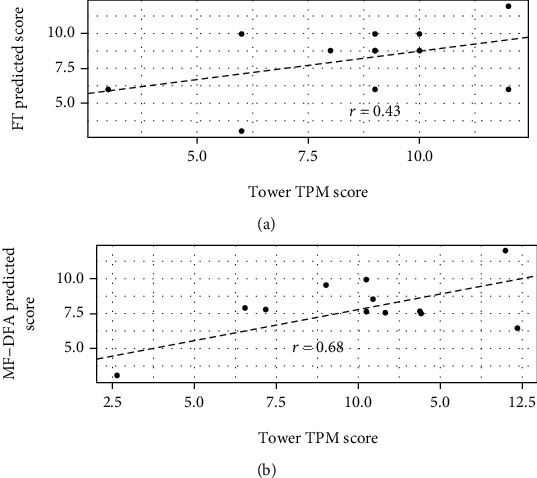
Correlations of FT- and MF-DFA EEG-derived CART model for TPM with actual TPM scores. Using all 64 leads and the first 30 s set of EEG parameters, a CART model was trained and then tested on the novel 30 s set. The resulting predicted scores are correlated with the actual TPM score for each participant: (a) FT-derived model; (b) MF-DFA-derived model. For demonstration purposes only, a small amount of noise was added to the TPM scores.

The MF-DFA test EEG-derived CART model correlated with actual Tower test TPM scores, whereas the FT CART model did not (Table 2 and Figure 1). However, the FT test EEG-derived CART model did correlate with actual Tower test RVPI scores, whereas the MF-DFA CART model did not (Table 2). Neither FT- nor MF-DFA test EEG-derived CART models correlated with Tower test ACH, FMT, MAR, or TRV subscores (Table 2).

### 3.3. CART-Derived Regression Models and Brain Regions: FT vs. MF-DFA

Regression models derived from CART data are diagrammed schematically in supplementary data (Figure S1). A summary of the relevant brain regions and statistical models generated is demonstrated in supplementary Table S1. For the FT-derived CART model for RVPI, lead 2 (lower right frontal), lead 30 (left temporal lobe), and lead 58 (lower right frontal) were included model parameters (Table S1).

For the MF-DFA-derived CART model for TPM, lead 1 (lower right frontal), lead 9 (middle left frontal), lead 24 (left temporal), and lead 52 (right temporal) were included model parameters (Table S1).

### 3.4. On a Reduced EEG Dataset, ITM Differs from FT on Ability to Predict Test EEG-Derived Tower Test Scores

**Figure 2:**
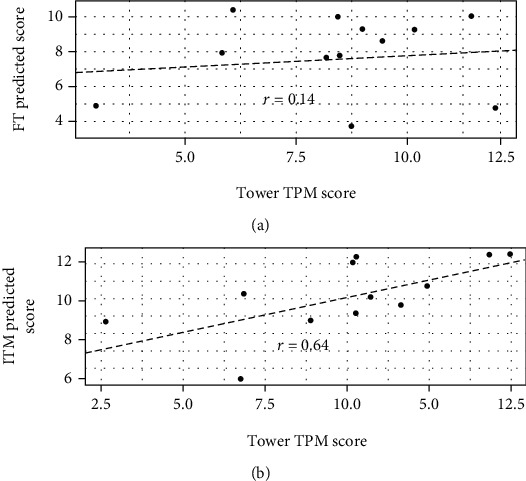
Correlations of FT and ITM EEG-derived CART model for TPM with actual TPM scores. Using the reduce 10 lead subsets and the first 30 s set of EEG parameters, a CART model was trained and then tested on the novel 30 s set. The resulting predicted scores are correlated with the actual TPM score for each participant: (a) FT-derived model; (b) ITM-derived model. For demonstration purposes only, a small amount of noise was added to the TPM scores.

Utilizing the reduced 10-lead EEG dataset, ITM test EEG-derived CART models correlated with actual Tower test ACH, FMT, MAR, RVPI, and TPM subscores (Table 3 and Figure 2). By contrast, the FT test reduced lead EEG-derived CART model only correlated with the actual Tower test RVPI scores (Table 3). Neither method's corresponding EEG-derived CART models correlated with TRV scores (Table 3).

### 3.5. CART-Derived Regression Models and Brain Regions for the Reduced Lead Set: FT vs. ITM

For the reduced lead FT-derived CART model for RVPI, lead 3 (middle right frontal) and lead 26 (left parietal/temporal) were included model parameters (supplementary Figure S1, supplementary Table S1). For the reduced-lead ITM-derived CART model for ACH, leads 6/56 (middle frontal to left frontal), lead 3 (middle right frontal), lead 31 (left parietal), lead 36 (midline parietal), and lead 40 (right parietal) were included parameters. For the ITM-derived CART model for MAR, lead 6 (middle frontal), leads 26/36 (left temporal to midline parietal), and lead 3 (middle right frontal) were parameters. For the ITM-derived CART model for FMT, leads 19/31 (left frontal to left parietal), lead 3 (middle right frontal), lead 36 (midline parietal), and lead 56 (lateral right frontal) were parameters. For RVPI, lead 3 (middle right frontal), lead 40 (right parietal), and lead 9 (middle left frontal) were CART model parameters. Finally, for TPM, leads 9/56 (middle left frontal to later left frontal), leads 3/6 (middle right frontal to midline frontal), lead 9 (middle left frontal), and lead 36 (midline parietal) were ITM-derived CART model parameters (Figure S1, Table S1).

## 4. Discussion

### 4.1. Differential Performance of MF-DFA and FT on the Ability to Predict Tower Test Executive Function Subscores from EEG

Given the dramatically different theoretical backgrounds behind FT and MF-DFA, it is perhaps not surprising that EEG-derived parameter models would prove to correlate with different aspects of Tower test performance (Table 2 and Figure 1). While TPM has been perhaps the best studied subscore of the Tower test [17], RVPI has been shown to specifically impaired in a small study of patients with focal lateral prefrontal cortex lesions [25]. Therefore, while MF-DFA-derived EEG in this study did correlate with the most widely used measure of executive function in the Tower test, there should certainly continue to be a role for FT, especially with regard to lesions with a propensity to RVPI impairment.

### 4.2. Differential Performance of ITM and FT and on the Ability to Predict Tower Test Executive Function Subscores from EEG

ITM-derived EEG parameters proved to be the most globally correlative with Tower test executive function subscores of the tests examined here, even in the reduced-lead paradigm (Figure 2 and Table 3). Indeed, ITM analysis only failed to correlate with Tower test TRV score amongst all subscores. By comparison, in the same reduced-lead subset, FT correlated only with RVPI (Table 3).

### 4.3. Potential General Utility of MF-DFA, ITM, and FT as Diagnostic Tools for Executive Function Deficits from EEG

ITM-derived EEG parameters clearly outperformed both MF-DFA and FT in this paradigm; in that in this study, they were able to correlate with most Tower test subscores. However, practically speaking, MF-DFA (in the case of TPM) and FT (in the case of RVPI) show promise in correlating with two of the most important subscores. It should be noted that the between-lead ITM analysis first described here is likely to represent a considerable improvement over within-lead ITM [6], as four of the five CART-derived correlation models incorporated between-lead analysis (Table S1). All this, it should be noted, while using a reduced set of leads. Future experience and more comprehensive analysis may allow for more complete EEG datasets to be successfully analyzed with ITM, which may further improve the accuracy of these models.

With regard to the TPM Tower test subscore, MF-DFA may continue to have an important role, given its efficacy (Figure 1 and Table 2). It is also likely to have the advantage of being relatively robust to noise, compared to other techniques to detect multifractality in time series' such as EEG recordings [26]. Indeed, even in the current study where a good proportion of the electrode recordings suffered from high levels of noise due to high impedance, MF-DFA produced a relatively accurate prediction of the Tower test TPM score from the test EEG (Table 2 and Figure 1). Ultimately, given that all three techniques proved to have some utility in correlating with Tower test subscores in an out-of-sample test, all three techniques should continue to be studied as potentially useful modalities for quantitative EEG research.

### 4.4. Temporal and Spatial Patterns of EEG-Based Correlates of Tower Test Performance

While far from a comprehensive study, the CART-based generation of linear models which associate with performance in Tower test subscores do provide some hints as to the localization of relevant brain regions (Figure S1 and Table S1). For instance, in both complete- and reduced-lead datasets, FT-derived EEG parameters for RVPI correlations find right frontal and left temporal beta spectral power are included in the model. These data substantially overlap with beta spectral power differences seen in a group of patients with mild blast-induced TBI (versus controls) via magnetoencephalography [27].

For the complete-lead dataset of MF-DFA-derived EEG parameters for the TPM correlation model, both left and right frontal and temporal lobe regions were included, with characteristic changes in region-specific minimum Hölder values (Figure S1 and Table S1). This indicates that variations in the amount of short-range correlation in EEG signals in frontal and temporal lobe leads are associated with performance changes in the Tower test TPM. Prior studies have demonstrated that reduced short-range and increased long-range correlation in EEG signals were associated with deep stages of sleep, compared to waking [4, 28]. Therefore, the assessment of changes in temporal correlations in EEG signals (e.g., via MF-DFA) may prove to be generally useful in studies of abnormal brain function, including TBI.

For the reduced-lead dataset of ITM-derived EEG parameters for the ACH correlation model, the middle frontal to left frontal mean information transfer constant ratio (*κ*_itcr_) at an 8 ms delay was an included model parameter, along with mean information transfer constants (*κ*) in the right frontal (4 ms), left (4 ms), right (32 ms), and middle (32 ms) parietal leads (Figure S1, Table S1). In the case of the FMT model, ITM-derived EEG parameters included left frontal to mid parietal at 3 s delay and midline parietal (4 ms) and right frontal (4 and 256 ms) values. For the MAR correlation model, ITM-derived EEG parameters included left temporal to midline parietal at 3 s and midline (4 ms) and right frontal (4 ms) values. The RVPI model ITM-derived EEG parameters contained left and right (4 ms each) frontal, along with right parietal (32 ms) values. Finally, the ITM-derived EEG parameter model for TPM included left frontal to lateral left frontal (64 ms) and right frontal to midline frontal (8 ms) values, along with left frontal (2 s) and midline parietal (4 ms) values. Therefore, EEG-based ITM-derived regression models for Tower test subscores provide both spatial and temporal data about brain regions involved in associative information processing. Indeed, the relevant timeframe can be as short as 4 milliseconds or as long at 3 seconds for between- or within-lead information transfer.

### 4.5. Limitations

The number of participants in this study was relatively small, and given the high dimensional nature of the parameterized EEG data, more definitive studies will await larger sample sizes in future studies. The population was limited to patients with mbTBI with no structural lesions, and therefore, these results may not apply to clinical populations with focal TBI or other gross structural brain pathologies. There was limited clinical information about concomitant medical or psychiatric history for the participants. Although BDI scores did not correlate with Tower test or MOCA performance, and none of the participants were taking psychotropic medications, some of the participants had elevated levels of depressive symptoms and/or histories of a diagnosis of major depression. Access to more comprehensive neuropsychological testing was not available; only MOCA and D-KEFS Tower test results were collected on the day of EEG collection. Information on the presence and severity of cognitive impairment is limited when using the MOCA with individuals with mild TBI. EEG data collection was hampered by poorly contacting electrodes in many cases, which may have impaired the study power overall.

## 5. Conclusions

The development of a biomarker derived from EEG signals which correlates with executive function deficits and associated neuroanatomical lesions would provide an important clinical tool for providers treating patients with TBI. Such an EEG-based biomarker for executive function deficits would be a major clinical innovation for treatment providers, given the high prevalence and associated morbidity of TBI. The current results are promising and merit further investigation of the ability of ITM, FT, and MF-DFA EEG analyses to provide an independent assessment of cognitive function. In particular, overall executive function (as assessed by TPM) seems to be best assessed via ITM and MF-DFA (Figures 1 and 2 and Tables 2 and 3), whereas RVPI is likely better assessed via FT or ITM (Tables 2 and 3). While based upon a limited sample, these data certainly give ample justification for larger studies of the potential for FT-, MF-DFA-, and ITM-based EEG analysis to correlate with executive function deficits in TBI.

## Figures and Tables

**Table 1:** Demographic and clinical characteristics of sample (*N* = 12).

Overall	Age	Male	White	Hispanic	BDI
	30.8 (2.7)	10 (83.3%)	9 (75%)	5 (41.7%)	21 (8.9)
Test component		Statistic vs. test component			
ACH	0.28	0.19	0.81	1.6	0.22
FMT	-0.35	0.29	0.2	1.4	-0.52
MAR	0.14	0.04	3.17	1.3	0.33
RVPI	0.54	1.4	2.5	0.17	-0.13
TPM	0.21	0.002	0.69	0.42	-0.08
Total score	0.18	0.2	0.67	0.07	-0.03

ACH: achievement score; FMT: first move time; MAR: TPM: time-per-move ratio. Listed statistics represent *r* values (Pearson's correlation for age and BDI) and *F*(1, 10) statistics (ANOVA for male, White, and Hispanic categories). None of the listed statistics result in statistical significance at the *p* < 0.05 threshold.

**Table 2:** Tower test out-of-sample EEG correlation coefficients with recursive partitioning: MF-DFA vs. FT.

Subscore	FT Corr	*t* stat	MF-DFA Corr	*t* stat
ACH	0.41	1.41	0.02	0.06
MAR	-0.011	-0.04	0.21	0.66
FMT	-0.08	-0.26	0.03	0.08
RVPI	*0.6*	*2.37* ^∗^	0.57	2.2
TPM	0.43	1.5	*0.68*	*2.94* ^∗^
TRV	0.34	1.14	0.31	1.03

ACH: achievement; MAR: mean accuracy ratio; FMT: first move time; RVPI: rule-violations-per-item ratio; TPM: time per move; TRV: total rule violations; Corr: Pearson's product-moment correlation value. Italic, ^∗^*p* < 0.05.

**Table 3:** Tower test out-of-sample EEG correlation coefficients with recursive partitioning: ITE vs. FT.

Subscore	FT Corr	*t* stat	ITE Corr	*t* stat
ACH	0.31	1.04	-0.19	-0.62
MAR	-0.33	-1.12	*0.92*	*7.25* ^∗^
FMT	0.2	0.63	*-0.72*	*-3.28* ^∗^
RVPI	*0.75*	*3.53* ^∗^	*0.71*	*3.15* ^∗^
TPM	0.14	0.44	*0.64*	*2.63* ^∗^
TRV	0.38	1.31	0.29	0.97

ACH: achievement; MAR: mean accuracy ratio; FMT: first move time; RVPI: rule-violations-per-item ratio; TPM: time per move; TRV: total rule violations; Corr: Pearson's product-moment correlation using reduced 10-lead EEG dataset. Italic, ^∗^*p* < 0.05.

## Data Availability

The data in the form of .csv spreadsheets will be available on a publicly accessible section of github.com upon acceptance of the manuscript for publication.

## Supplementary Materials

Supplementary 1 Supplementary Figure 1: classification tree plots for EEG parameter models with significant correlations with Tower test subscores. As in Figures 1 and 2, these plots represent CART-derived tree plots of the actual successful correlation models. Each tree branch is described by the lead number, followed by the classifier after a period, then an operator, then the classifying value. For example, “L30.bet < 48e+3” indicates “lead 30 beta power < 4800” as the classifying value in the first panel. FT: alp- alpha; bet- beta power. MF-DFA: avgh- average Holder value; minh- minimum Holder value. ITM: *V* signifies the lead, with the number after the period indicating the time step Δ*t* (in units of sampling rate 250 Hz), with the classifier as the value of the mean Information Transfer Constant *κ* at the time step. Classifiers with two leads listed (e.g., “V6V52”) indicates the value of the relevant Information Transfer Constant Ratio *κ*ITCR, again with the number after the period indicating the time step in units of the sampling rate.

Supplementary 2 Table 1: CART-derived parameter rules from successful models.
