# Supplementary material for: Information Transfer and Multifractal Analysis of EEG in Mild Blast-Induced TBI
Source: Comput Math Methods Med. 2021 Apr 6;2021:6638724. doi: 10.1155/2021/6638724 (PMC8051525; doi:10.1155/2021/6638724)

**Supplementary Material**

**Supplementary Figure 1. Classification tree plots for EEG parameter models with significant correlations with Tower test subscores.** As in Figures 1 and 2, these plots represent CART-derived tree plots of the actual successful correlation models. Each tree branch is described by the lead number, followed by the classifier after a period, then an operator, then the classifying value. For example, “L30.bet < 48e+3” indicates “lead 30 beta power < 4800” as the classifying value in the first panel.

FT: **alp**- alpha; **be**t- beta power.

MF-DFA: **avgh**- average Holder value; **minh**- minimum Holder value.

ITM: V signifies the lead, with the number after the period indicating the time step Δt (in units of sampling rate 250 Hz), with the classifier as the value of the mean Information Transfer Constant κ at the time step. Classifiers with two leads listed (e.g. “V6V52”) indicates the value of the relevant Information Transfer Constant Ratio κ_ITCR_, again with the number after the period indicating the time step in units of the sampling rate.

**Supplementary Figure 1**


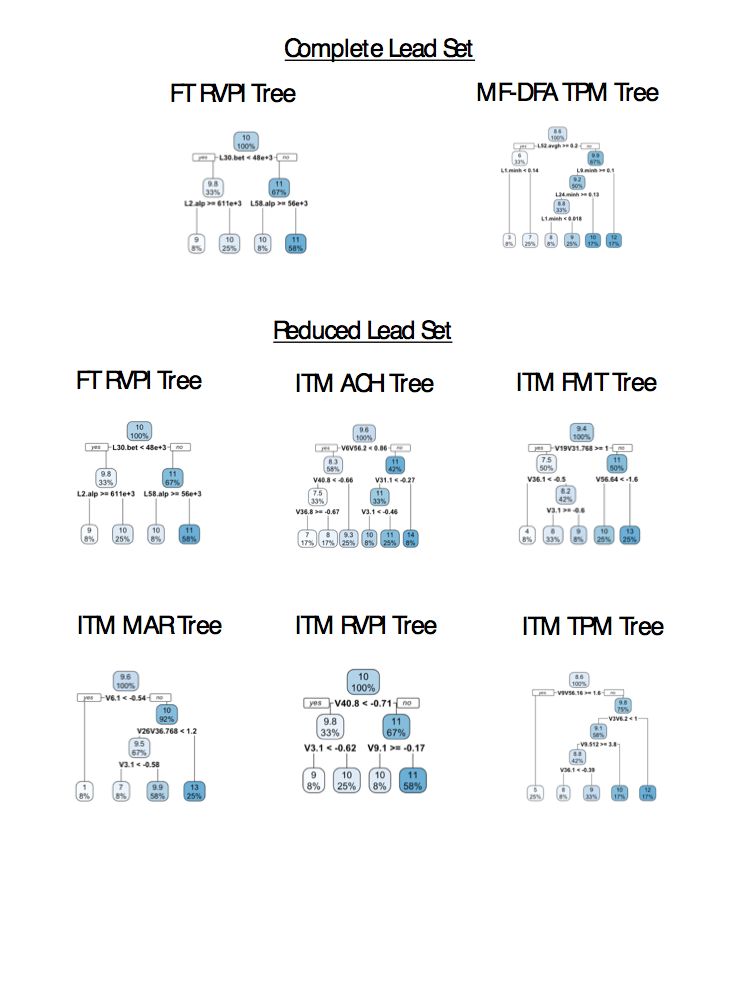

Supplement: Supplementary 1 — Supplementary Figure 1: classification tree plots for EEG parameter models with significant correlations with Tower test subscores. As in Figures 1 and 2, these plots represent CART-derived tree plots of the actual successful correlation models. Each tree branch is described by the lead number, followed by the classifier after a period, then an operator, then the classifying value. For example, “L30.bet < 48e+3” indicates “lead 30 beta power < 4800” as the classifying value in the first panel. FT: alp- alpha; bet- beta power. MF-DFA: avgh- average Holder value; minh- minimum Holder value. ITM: V signifies the lead, with the number after the period indicating the time step Δt (in units of sampling rate 250 Hz), with the classifier as the value of the mean Information Transfer Constant κ at the time step. Classifiers with two leads listed (e.g., “V6V52”) indicates the value of the relevant Information Transfer Constant Ratio κITCR, again with the number after the period indicating the time step in units of the sampling rate. [file 6638724.f1.docx]
