# Supplementary material for: Information Transfer and Multifractal Analysis of EEG in Mild Blast-Induced TBI
Source: Comput Math Methods Med. 2021 Apr 6;2021:6638724. doi: 10.1155/2021/6638724 (PMC8051525; doi:10.1155/2021/6638724)
Supplement: Supplementary 2 — Table 1: CART-derived parameter rules from successful models. [file 6638724.f2.pdf]

Supplementary Table 1: CART-derived parameter rules from successful models

All lead data

Technique	Subscore	CART correlation model rules	Brain regions and variables
**FT**	**RVPI**	9 when L30.bet < 47727 & L2.alp >= 610918<br>10 when L30.bet < 47727 & L2.alp < 610918<br>10 when L30.bet >= 47727 & L58.alp >= 56373<br>11 when L30.bet >= 47727 & L58.alp < 56373	lower right frontal alpha<br>lower right frontal beta<br>left temporal beta
**MF-DFA**	**TPM**	3 when L52.avgh >= 0.2 & L1.minh < 0.135<br>7 when L52.avgh >= 0.2 & L1.minh >= 0.135<br>8 when L52.avgh < 0.2 & L1.minh < 0.018 & L9.minh >= 0.1 &<br>9 when L52.avgh < 0.2 & L1.minh >= 0.018 & L9.minh >= 0.1 & L24.minh >= 0.13<br>10 when L52.avgh < 0.2 & L9.minh >= 0.1 & L24.minh < 0.13<br>12 when L52.avgh < 0.2 & L9.minh < 0.1	lower right frontal minimum h value<br>middle left frontal minimum h value<br>left temporal minimum h value<br>right temporal average h value

Reduced lead set data

Technique	Subscore	CART correlation model rules	Brain regions and variables
**FT**	**RVPI**	9.7 when L3.bet >= 124797<br>10.0 when L3.bet < 124797 & L26.bet < 34656<br>11.0 when L3.bet < 124797 & L26.bet >= 34656	middle right frontal beta<br>left parietal/temporal beta
**ITM**	**ACH**	7.0 when L6L56.2 < 0.86 & L40.8 < -0.66 & L36.8 >= -0.67<br>8.0 when L6L56.2 < 0.86 & L40.8 < -0.66 & L36.8 < -0.67<br>9.3 when L6L56.2 < 0.86 & L40.8 >= -0.66<br>10.0 when L6L56.2 >= 0.86 & L31.1 < -0.27 & L3.1 < -0.46<br>11.0 when L6L56.2 >= 0.86 & L31.1 < -0.27 & L3.1 >= -0.46<br>14.0 when L6L56.2 >= 0.86 & L31.1 >= -0.27	middle frontal to left frontal 8 ms kappa ITCR<br>middle right frontal at 4 ms kappa<br>left parietal at 4 ms kappa<br>right parietal at 32 ms kappa<br>midline parietal at 32 ms kappa
	**MAR**	1.0 when L6.1 < -0.54<br>7.0 when L6.1 >= -0.54 & L26L36.768 < 1.2 & L3.1 < -0.58<br>9.9 when L6.1 >= -0.54 & L26L36.768 < 1.2 & L3.1 >= -0.58<br>12.7 when L6.1 >= -0.54 & L26L36.768 >= 1.2	midline frontal at 4 ms kappa<br>left temporal to midline parietal at 3 s kappa ITCR<br>middle right frontal at 4 ms kappa
	**FMT**	4 when L19L31.768 >= 1 & L36.1 < -0.5<br>8 when L19L31.768 >= 1 & L36.1 >= -0.5 & L3.1 >= -0.6<br>9 when L19L31.768 >= 1 & L36.1 >= -0.5 & L3.1 < -0.6<br>10 when L19L31.768 < 1 & L56.64 < -1.6<br>13 when L19L31.768 < 1 & L56.64 >= -1.6	left frontal to left parietal at 3 s kappa ITCR<br>midline parietal at 4 ms kappa<br>middle right frontal at 4 ms kappa<br>lateral right frontal at 256 ms kappa
	**RVPI**	9 when L40.8 < -0.71 & L3.1 < -0.62<br>10 when L40.8 < -0.71 & L3.1 >= -0.62<br>10 when L40.8 >= -0.71 & L9.1 >= -0.17<br>11 when L40.8 >= -0.71 & L9.1 < -0.17	middle right frontal at 4 ms kappa<br>right parietal at 32 ms kappa<br>middle left frontal at 4 ms kappa
	**TPM**	5 when L9L56.16 >= 1.6<br>8 when L9L56.16 < 1.6 & L3L6.2 < 1 & L9.512 >= 3.8 & L36.1 < -0.39<br>9 when L9V56.16 < 1.6 & L3L6.2 < 1 & L9.512 >= 3.8 & L36.1 >= -0.39<br>10 when L9L56.16 < 1.6 & L3L6.2 < 1 & L9.512 < 3.8<br>12 when L9L56.16 < 1.6 & L3L6.2 >= 1	middle left frontal to lateral left frontal at 64 ms kappa ITCR<br>middle right frontal to midline frontal at 8 ms kappa ITCR<br>middle left frontal at 2 s kappa<br>midline parietal at 4 ms kappa
